# Draft Genome Sequence of the Filamentous Fungus Penicillium paxilli (ATCC 26601)

**DOI:** 10.1128/genomeA.00071-15

**Published:** 2015-03-12

**Authors:** Daniel Berry, Murray P. Cox, Barry Scott

**Affiliations:** Institute of Fundamental Sciences, Massey University, Palmerston North, New Zealand

## Abstract

Penicillium paxilli ATCC 26601 is an asexual filamentous fungal species known for its production of the mycotoxin paxilline. We present here the 35-Mb draft genome sequence for this organism.

## GENOME ANNOUNCEMENT

Penicillium paxilli is an asexual saprophytic fungus, and isolate ATCC 26601 originates from insect-damaged pecans in Georgia, USA (1). P. paxilli is known for its production of the indole-diterpene paxilline, a potent tremorgen (1) that inhibits calcium-activated potassium channels (2). Due to the high rate of growth, sporulation, and ease of genetic modification, P. paxilli has been utilized as a model organism for studying indole-diterpene biosynthesis (3–8). The paxilline biosynthetic machinery is encoded by the *PAX* gene cluster, and the promoter from the *paxM* gene of this cluster has been successfully used for the heterologous expression of indole-diterpene biosynthetic genes from the fungal grass endophyte *Epichloë* festucae (9). The genome sequence of P. paxilli was sequenced to investigate its suitability as a heterologous expression system for fungal nonribosomal peptide synthetases (NRPS).

P. paxilli genomic DNA was prepared by phenol-chloroform extraction (10) and treated with RNase A. Two runs with 150-bp paired-end fragment reads were done on an Illumina MiSeq instrument by New Zealand Genomics Limited (NZGL), obtaining approximately 182-fold genome coverage. The reads were dynamically trimmed using the SolexaQA package to their longest fragment, such that the base call error rates did not exceed a *P* value of 0.05, and paired-end reads of <100 bp were discarded. *De novo* assembly was performed on all data using ABySS version 1.3.0, with the parameters *n* = 2, c = 10, and *k* = 79. The final assembly consisted of 635 contigs, with 414 contigs >500 bp in size, an average contig length of 84,079 bp, a maximum contig length of 732,567 bp, an *N*_50_ of 189,821 bp, and a total of 34,808,516 residues.

Blast+ was used to identify a 4′-phosphopantetheinyl transferase (DDBJ/EMBL/GenBank accession no. KP233470) and a type II thioesterase (accession no. KP233471) in the P. paxilli genome that are likely to be functional. These proteins are required to produce and maintain functional NRPS enzymes, indicating that P. paxilli has the potential to be a suitable heterologous expression system for fungal NRPSs.

### Nucleotide sequence accession numbers.

This whole-genome shotgun project has been deposited at DDBJ/EMBL/GenBank under the accession no. AOTG00000000. The version described in this paper is the first version, AOTG01000000.
